# Genotype-Guided Use of P2Y12 Inhibitors: A Review of Current State of the Art

**DOI:** 10.3389/fcvm.2022.850028

**Published:** 2022-03-23

**Authors:** Abdullah Al-abcha, Yasser Radwan, Danielle Blais, Ernest L. Mazzaferri, Konstantinos Dean Boudoulas, Essa M. Essa, Richard J. Gumina

**Affiliations:** ^1^Division of Internal Medicine, Department of Medicine, Michigan State University, East Lansing, MI, United States; ^2^Division of Cardiology, Department of Medicine, Ohio State University, Columbus, OH, United States; ^3^Division of Cardiology, Department of Medicine, Mount Carmel Healthcare, Columbus, OH, United States

**Keywords:** genotype, P2Y12, guided therapy, platelet, function

## Abstract

The pharmacodynamics of the purinergic receptor type Y, subtype 12 (P2Y12) inhibitors has evolved. Our understanding of the metabolism of P2Y12 inhibitors has revealed polymorphisms that impact drug metabolism and antiplatelet efficacy, leading to genetic testing guided therapy. In addition, assays of platelet function and biochemistry have provided insight into our understanding of the efficacy of “antiplatelet” therapy, identifying patients with high or low platelet reactivity on P2Y12 therapy. Despite the data, the implementation of these testing modalities has not gained mainstream adoption across hospital systems. Given differences in potency between the three clinically available P2Y12 inhibitors, the balance between thrombotic and bleeding complications must be carefully considered, especially for the large proportion of patients at higher risk for bleeding. Here we review the current data for genetic and functional testing, risk assessment strategies, and guidelines for P2Y12 inhibitors guided therapy.

## Introduction

According to international guidelines, dual antiplatelet therapy (DAPT) consisting of aspirin and a P2Y12 inhibitor is indicated in patients with coronary artery disease (CAD) treated with percutaneous coronary intervention (PCI) (1). When compared to clopidogrel, treatment with more potent P2Y12 inhibitors (ticagrelor or prasugrel) are associated with a lower incidence of recurrent thrombotic events and a higher bleeding risk (1–3). The more potent P2Y12 inhibitors are the first line agents in patients with acute coronary syndrome (ACS) undergoing PCI, however in cases of contraindications, clopidogrel is recommended (4). On the other hand, the American College of Cardiology (ACC) recommends the use of clopidogrel as the preferred antiplatelet agent after PCI in patients requiring long-term oral anticoagulation (5).

Clopidogrel is a prodrug that requires bioactivation by cytochrome P450 (CYP) to its active metabolite. Studies have shown patients with CYP loss of function (LOF) alleles have higher risk of major adverse cardiovascular events (MACE) especially stent thrombosis when treated with clopidogrel compared to non-carriers (6). Here we review the studies, observational and randomized clinical trials (RCTs), that have investigated a genetic and functional testing guided approach to antiplatelet therapy in patients treated with PCI.

## Pharmacology and Genetics of P2Y12 Inhibitors

**Figure 1:**
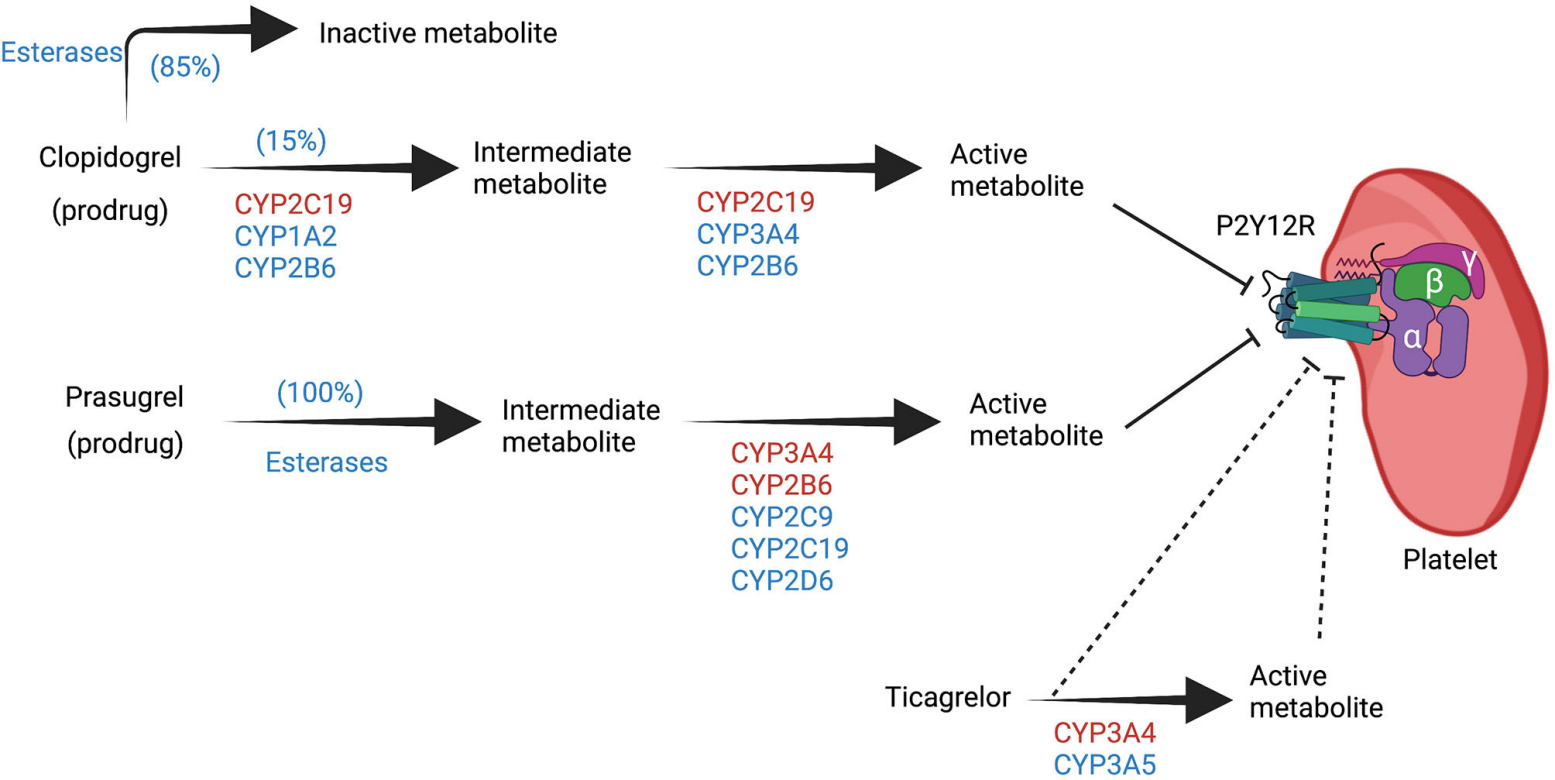
Overview of the biotransformation of P2Y12 inhibitors. Clopidogrel and prasugrel are prodrugs that require bioactivation *via* cytochrome P450 (CYP) to their active metabolite that can inhibit the P2Y12 receptor in a competitive manner. The two steps of bioactivation of clopidogrel are CYP dependent, while only one step of prasugrel bioactivation is CYP dependent. Ticagrelor undergoes biotransformation to another metabolite via CYP but both ticagrelor and its metabolite can inhibit the P2Y12 receptor in a non-competitive manner. CYP enzymes highlighted in red have more significant roles in each of the steps. Created with BioRender.com. CYP, cytochrome P450, P2Y12 R, P2Y12 receptor.

P2Y12 receptor is a platelet membrane protein that is coupled to Gi protein (7) (Figure 1). The damage of cells, and platelets result in the release of adenosine diphosphate (ADP). ADP binds to the P2Y12 receptors resulting in platelet activation, exposing activated glycoprotein IIb/IIIa (GPIIb/IIIa), and P-selectin that drive further platelet aggregation and recruitment resulting in stabilization of thrombus formation (7). While, GPIIb/IIIa antagonists were first developed as potent inhibitors of this process, subsequent work has demonstrated inhibition of P2Y12 is a crucial step in the prevention of thrombus propagation. The development of P2Y12 specific inhibitors has provided robust pharmacologic options. To date, six P2Y12 inhibitors have been developed for clinical use (Table 1). Thienopyridines including ticlopidine, clopidogrel, and prasugrel are prodrugs that require bioactivation to their active metabolites. Once activated, they irreversibly bind to P2Y12 receptors preventing the binding of ADP and inhibit further platelet activation (8). The non-thienopyridines include ticagrelor, cangrelor, and selatogrel. Ticagrelor is a reversible non-competitive inhibitor, while cangrelor and selatogrel are direct reversible inhibitors of the P2Y12 receptor.

**Table 1:** Overview of the P2Y12 inhibitors.

	**Thienopyridines**			**Non-thienopyridines**		
	**Clopidogrel**	**Prasugrel**	**Ticlopidine**	**Ticagrelor**	**Cangrelor**	**Selatogrel**
Route of administration	Oral	Oral	Oral	Oral	Intravenous	Subcutaneous
Bioactivation	Yes	Yes	Yes	No	No	No
Mechanism of action	Competitive inhibition of P2Y12 receptor	Competitive inhibition of P2Y12 receptor	Competitive inhibition of P2Y12 receptor	Non-competitive inhibition of P2Y12 receptor	Competitive inhibition of P2Y12 receptor	Competitive inhibition of P2Y12 receptor
Reversibility	Irreversible	Irreversible	Irreversible	Reversible	Reversible	Reversible
Half life	6 h	7 h	13 h	6–12 h	3–6 min	4–7 h
Onset of action	2–8 h	0.5–4 h	6 h	0.5–4 h	2 min	15–30 min

### Thienopyridines

Ticlopidine was the first commercially available oral agent from the thienopyridines class. It required a two-step CYP-dependent hepatic bioactivation. CYP2B6, CYP2C19, and CYP3A are the major CYP isoforms involved in its bioactivation. Before ticlopidine was removed from the market, one side effect limiting its use was neutropenia.

**Figure 2:**
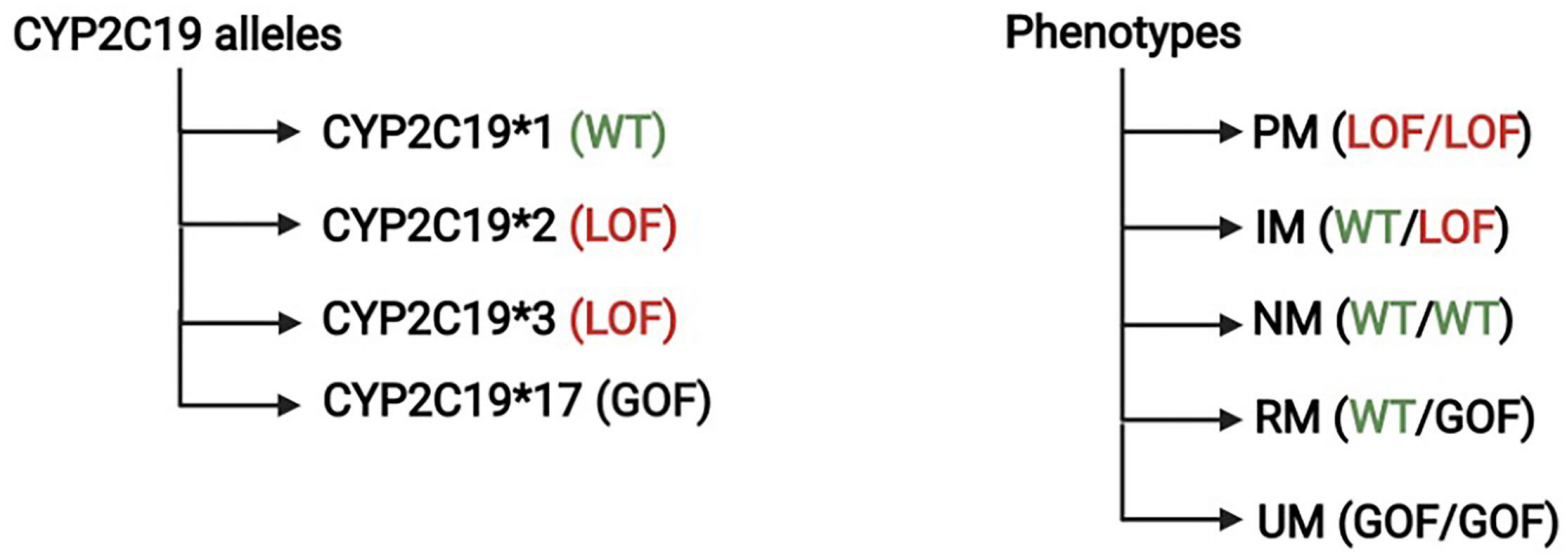
Cytochrome P450 genotypes and phenotypes. The Clinical Pharmacogenetics Implementation Consortium categorized the level of CYP2C19 function into five different phenotypes; poor metabolizers (PM), intermediate metabolizers (IM), normal metabolizers (NM), rapid metabolizers (RM), and ultrarapid metabolizers (UM).

Clopidogrel requires a two-step CYP-dependent hepatic bioactivation as well. Similar to ticlopidine, 15% of the oral clopidogrel dose gets activated by hepatic CYP, while 85% are inactivated by esterases and subsequently excreted. CYP2C19 is the most influential isoform in the bioactivation processes, and the gene responsible for its expression is located on chromosome 10. CYP2C19-1 is the wild-type allele with normal enzymatic function. Genetic polymorphism results in different alleles with different degree of enzymatic function that range from complete LOF to increased activity and gain of function (9). CYP2C19–2, –3, and –17 are the most common genetic variations; CYP2C19–2, –3 are the LOF alleles, while CYP2C19-17 have increased enzymatic activity (9). A consensus statement from the Clinical Pharmacogenetics Implementation Consortium (CPIC) categorized individuals into five different phenotypes according to their alleles; poor metabolizers (PM), intermediate metabolizers (IM), normal metabolizers (NM), rapid metabolizers (RM), and ultrarapid metabolizers (UM) (Figure 2) (10).

Prasugrel requires CYP bioactivation as well. It is activated by similar CYP isoforms as clopidogrel but CYP2B6, and CYP3A4 are the most influential isoforms with a smaller role of CYP2C19 (11). Unlike clopidogrel, there is no inactivation pathway, and esterase plays a role in the activation process of prasugrel (Figure 1).

#### Non-thienopyridines

Ticagrelor is an oral cyclopentyl-triazolopyrimidines that reversibly binds P2Y12 receptor in a non-competitive manner resulting in a conformational change that limits the receptor's ADP binding capacity (12, 13). Ticagrelor does not require bioactivation and it is metabolized by CYP3A4 into an active metabolite with comparable antiplatelet activity as ticagrelor (14).

Cangrelor is a non-thienopyridine, direct ATP analog that reversibly binds to the P2Y12 receptor. It is the only intravenous P2Y12 receptor inhibitor that has a rapid onset of action and undergoes rapid hydrolysis which results in a half-life of 3–6 min (15). Cangrelor has a unique clinical niche specifically for use in patients with ACS undergoing PCI and has not received an oral P2Y12 inhibitor or when the use of oral agent is not preferred including patients who are in cardiogenic shock or those without enteral access (15); we will not discuss studies using cangrelor in this review.

Selatogrel is a novel non-thienopyridine that is administered subcutaneously. Phase 1 and 2 studies have shown that the medication has a rapid onset of action, reversibly inhibits P2Y12 receptors and has an acceptable safety profile (16). Phase 3 trials are being developed to assess its impact on clinical outcomes; we will not discuss studies using selatogrel in this review (17).

## Implications of Genetic Polymorphism

Genetic polymorphisms play a significant role in pharmacokinetics, pharmacodynamics, and clinical efficacy of clopidogrel. The frequencies of CYP2C19 alleles varies between race/ethnic groups (18). CYP2C19-1 is the most common allele found across different ethnicities (58% in African Americans, 62% in Caucasians, and 58% in East Asians. CYP2C19-2 is more prevalent in the East Asian population compared to African Americans and Caucasians (East Asians 29%, African Americans 18%, and Caucasians 14.6%) while CYP2C19-17 is more common in Caucasian populations (Caucasians 22%, African Americans 19%, and East Asians 1.6%) (18).

In 162 healthy volunteers who were given a loading dose of clopidogrel, patients were determined to be PM or IM by carrying one or two of CYP2C19 LOF alleles. Compared to non-carries, PM or IM patients had more than 30% relative reduction in plasma exposure to active metabolite (*p* < 0.001) and more than 20% relative reduction in maximal platelet aggregation from baseline (*p* < 0.001) (19). In a *post-hoc* analysis of 1,477 patients who received clopidogrel in the TRITON-TIMI 38 trial), CYP2C19 PM or IM patients had higher rate of MACE when compared to non-carriers (11.7 vs. 8.3%, *p* = 0.04) (19). No clinical significance was achieved in patients with LOF of CYP2C9, CYP2B6, CYP3A5, or CYP1A2. Sibbing et al. performed genetic analysis on 2,485 patients pretreated with clopidogrel and subsequently underwent PCI. Patients with at least one CYP2C19 LOF allele had significantly higher rate of stent thrombosis at 30 days when compared to non-carriers (1.5 vs. 0.4%, *p* = 0.007) (20).

The clinical role of CYP2C19 gain of function alleles (CYP2C19-17) is less defined. Tiroch et al. reported a protective role of CYP2C19 gain of function alleles as carriers of CYP2C19-17 had a lower risk of target-lesion revascularization and MACE when compared to non-carriers (21). However, Lee et al. reported a similar risk of MACE between carriers of CYP2C19-17and non-carrier (22). In terms of bleeding risk, Sibbing et al. reported increased bleeding risk in carriers of CYP2C19-17 (23), while Lee et al. reported similar bleeding risk between carriers and non-carriers (22).

Compared to clopidogrel, genetic polymorphism has a minor role in the metabolism of prasugrel or ticagrelor and does not affect their clinical efficacy. Mega et al. compared the pharmacokinetics, and pharmacodynamics of prasugrel in 238 healthy volunteers between CYP LOF alleles carriers (CYP2B6, CYP3A4, or CYP2C19) vs. non-carriers. There was no difference in active metabolite levels, or inhibition of platelet aggregation between the two group (11). In a *post-hoc* analysis of 1,466 patients who received prasugrel in the TRITON-TIMI 38 trial, there was no difference in risk of MACE between carriers of CYP LOF alleles vs. non-carriers (11). Varenhorst et al. performed genetic analysis on patients who received ticagrelor in the PLATO trial. Three genetic alleles (CYP3A4, UGT2B7, and SLCO1B1) were identified and affected ticagrelor pharmacokinetics, but this change had no clinical significance as MACE was similar between carriers and non-carriers of these alleles (24). There is limited data regarding the role of CYP polymorphism in ticlopidine metabolism as the drug has been removed from the market due to its significant side effect profile including neutropenia compared to the newer available agents (25).

The critical point is that the predominant genetic polymorphism that affect clinical outcomes are the CYP2C19 LOF alleles (CYP2C19^*^2, and ^*^3) in patients receiving clopidogrel.

## Impact of Platelet Reactivity on Clinical Outcomes

### Measures of Platelet Reactivity

**Figure 3:**
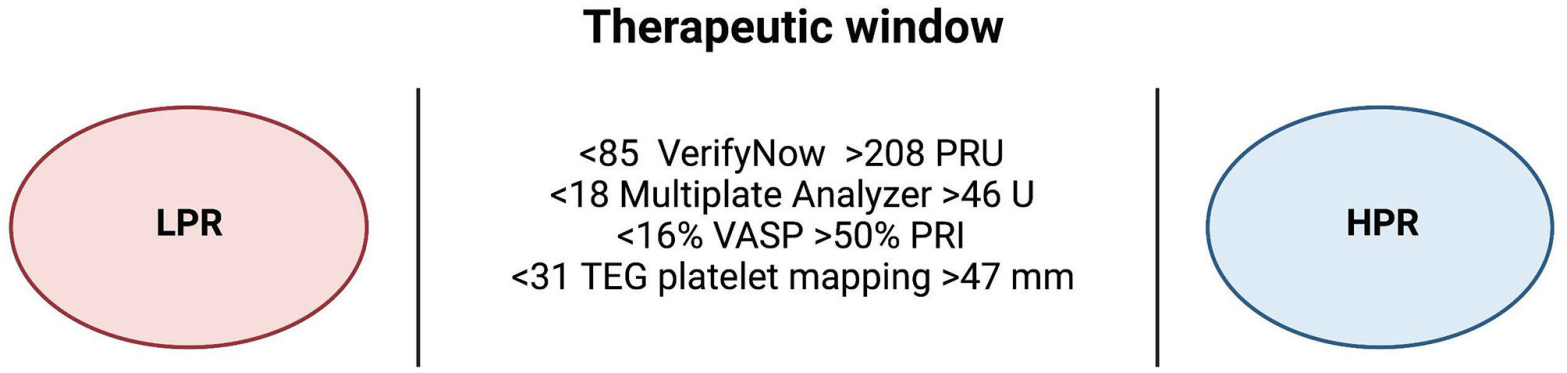
Therapeutic window of different platelet function testing assays. An expert consensus statement has defined the therapeutic window of platelet reactivity using different platelet function testing assays. Measurements higher than the therapeutic window are defined as high platelet reactivity (HPR), and patients with HPR are associated with high ischemic risk while measurements lower than the therapeutic window are defined as low platelet reactivity (LPR), and patients with LPR are at high bleeding risk. Created with BioRender.com. HPR, high platelet reactivity; LPR, low platelet reactivity; PRU, platelet reactivity unit; PRI, platelet reactivity index; TEG, thromboelastography; U, unit; VASP, vasodilator-stimulated phosphoprotein.

Platelet function testing (PFT) can be conducted with multiple available assays (Figure 3). These assays can be classified as laboratory-based vs. point-of-care assays. Laboratory-based assays include light transmittance aggregometry and vasodilator-stimulated phosphoprotein (VASP). Light transmittance aggregometry is an optical detection system. It utilizes an agent to induce platelet aggregation and then measures the changes in turbidity to detect the degree of platelet aggregation (26). VASP is an intracellular regulatory protein that is considered a marker of P2Y12 reactivity. P2Y12 stimulation results in VASP dephosphorylation while P2Y12 inhibition results in VAST phosphorylation, thus VASP assay utilizes detection of VSAP phosphorylation/dephosphorylation *via* flow cytometry to measure response to P2Y12 inhibitors (27).

Point-of-care assays include VerifyNow, Multiplate analyzer (MAP), and thromboelastography (TEG) with platelet mapping. These assays are preferred over the laboratory-based assays in clinical practice (28). The VerifyNow assay utilizes a whole blood sample to measure light transmittance after the aggregation of platelets to fibrinogen coated beads (29). MAP is an impedance aggregometer that measures change in impedance after the binding of platelets to electrodes as a response to an agonist (arachidonic acid, collagen, or ADP) (30). TEG with platelet mapping assay measures changes in the viscoelasticity of blood. It provides global assessment of hemostasis, as well as the speed of clot formation, stability, and degradation. It also provides assessment of platelets' role in the formation of the clots as it incorporates all the components of coagulation including clotting factors, fibrin and thrombin (31).

#### Studies of Platelet Reactivity

Pharmacological studies have shown higher platelet reactivity (HPR) measured by PFT assays in patients on clopidogrel when compared to the other P2Y12 inhibitors (32, 33). A randomized pharmacological study assessed platelet reactivity between patients on prasugrel vs. switching patients from prasugrel to ticagrelor with and without loading dose. Platelet inhibition transiently decreased in the ticagrelor group irrespective of receiving a loading dose with no HPR in either group (34). Genetic polymorphism and LOF-alleles play a significant role in platelet reactivity, specifically HPR in patients receiving antiplatelet therapy (35–37). Zhang et al. conducted a randomized pharmacological study where patients on clopidogrel were randomized into two arms; a control arm where all patients were continued on clopidogrel 75 mg once daily vs. genotype-guided arm where patients underwent genotype testing for CYP2C19^*^2, and ^*^3 and PM switched to ticagrelor, IM switched to clopidogrel 75 mg twice daily, and NM continued clopidogrel 75 mg once daily. After 5 days, platelet reactivity was measured using TEGs, and patients with HPR were significantly lower in the guided arm (29.6%) when compared to the control arm (38.1%, *p* < 0.001) (38).

HPR is considered a marker of higher ischemic risk in patients on antiplatelets therapy (39–41). The ADAPT-DES study was a prospective study that assessed the relation between HPR and clinical outcomes in patients receiving clopidogrel. HPR was detected using the VerifyNow assay and had a cut off >208 platelet reactivity unit (PRU). At 1 year, the risk of stent thrombosis, myocardial infarction, and clinically relevant bleeding were all higher in patients with HPR when compared to patients with no HPR (41). A recent analysis of the ADAPT-DES study was based on the clinical presentation and platelet reactivity (42). At 30 days, the risk of stent thrombosis was significantly higher in patients with HPR presenting with MI (1.8%) when compared to patients with HPR presenting with non-ACS (0.5%), patients with no HPR presenting with MI (0.3%), and patients with no HPR presenting with non-ACS (0.2%). This emphasizes the importance of clinical presentation in addition to platelet reactivity in patients receiving antiplatelet therapy (42).

Ethnic and racial groups have different responses to P2Y12 inhibitors and different on-treatment platelet reactivity. LOF alleles and HPR are more prevalent in the East Asian population (18, 43). Despite this, multiple studies have reported lower incidence of ischemic outcomes in the East Asian population when compared to the Caucasian population which has been described as the “East Asian paradox” (43).

### Evidence for the Use of Platelet Reactivity Guided P2Y12 Inhibitor Selection

Multiple major randomized and non-randomized studies have been conducted to investigate the efficacy and safety of PFT guided anti-platelets therapy (Table 2). Bonello et al. conducted the first RCT to evaluate VASP-guided antiplatelet therapy (44). They included 162 patients who underwent PCI and had HPR after a loading dose of 600 mg of clopidogrel (VSAP > 50%) and randomized them to control arm with no additional clopidogrel doses and the VASP-guided arm where patient received up to three additional loading doses of 600 mg of clopidogrel to lower than VASP <50%. At 1 month of follow up, MACE was significantly lower in the VASP arm (0%) when compared to the control (10%, *p* = 0.007), and the rate of major and minor bleeding was similar between the two arms (VASP 5% vs. Control 4%, *p* = 1) (44). The GRAVITAS study was a RCT that included patients with HPR measured by VerfiyNow undergoing PCI (47). A cutoff of ≥230 PRU was used to determine HPR. Patients were randomized to either receive high dose clopidogrel (loading with 600 mg and maintenance of 150 mg) vs. low dose of 75 mg maintenance with no loading dose. At 6 months, the rate of MACE was similar between the two arms (high-dose 2.3% vs. low dose 2.3%, *p* = 0.97) as well as the rate of major bleeding (high-dose 1.4% vs. low dose 2.3%, *p* = 0.10) (47).

**Table 2:** Summary of all studies that compared clinical outcomes based on platelet function testing-guided P2Y12 inhibitors.

	**Population**	**Patients with ACS**	**Platelet function assay and its cutoff**	**Control arm**	**Experimental arm**	**Follow up duration in months**	**Results**
**Randomized clinical trials**							
Bonello et al. (44)	162	46.2%	VASP, HPR: VASP > 50%	HPR patients received clopidogrel (loading dose 600 mg)	VASP-guided; HPR patients received clopidogrel loading dose of 600 mg and up to 3 additional loading doses of 600 mg to achieve VASP <50%	1	- MACE was significantly lower in the VASP arm (VASP 0% vs. control 10%, *p* = 0.007) - Major bleeding events were similar between the two arms (VASP 5% vs. control 4%, *p* = 1)
Bonello et al. (45)	429	51.5%	VASP, HPR: VASP > 50%	HPR patients received clopidogrel (loading dose 600 mg)	VASP-guided; HPR patients received clopidogrel loading dose of 600 mg and up to 3 additional loading doses of 600 mg to achieve VASP <50%	1	- MACE was significantly lower in the VASP-guided arm (VASP-guided 0.5% vs. control 8.9%, *P* <0.001) - Major and minor bleeding rate was similar between the two arms (VASP-guided 3.7% vs. control 2.8%, *p* = 0.8)
Wang et al. (46)	306	20%	VASP, HPR: VASP >50%	HPR patients received clopidogrel (300 mg loading dose and 75–375 mg maintenance dose)	HPR patients received the same loading dose of clopidogrel but their maintenance dose was adjusted throughout the follow up period to keep VASP <50%	12	- MACE was significantly lower in the VASP-guided arm (VASP-guided 9.3% vs. control 20.4%, *p* = 0.008) - Major and minor bleeding rate was numerically lower in the VASP arm without statistical significance (VASP-guided 12.9% vs. control 16.6%, *p* = 0.06)
GRAVITAS (47)	2,214	39.8%	VerifyNow, HPR: ≥230 PRU	HPR patients received clopidogrel 75 mg maintenance dose without loading	HPR patients received clopidogrel with 600 mg loading dose and 150 mg maintenance dose	6	- MACE was similar between the two arms (high-dose 2.3% vs. low dose 2.3%, *p* = 0.97) - Major bleeding events were similar between the two arms (high-dose 1.4% vs. low dose 2.3%, *p* = 0.10)
Bonello (48)	301	100%	VASP, HPR: VASP >53.5% LPR: VASP ≤ 16%	All patients were on prasugrel, and divided into 1. Patients without HPR 2. Patients with HPR 3. Patients without LPR 4. Patients with LPR		12	- Thrombotic evens were significantly higher in patients with HPR (22.4%) when compared to patients without HPR (2.9%, *p* < 0.001) - Bleeding events were significantly higher in patients with LPR (15.6%) when compared to patients without LPR (3.3%, *p* < 0.001)
Aradi (49)	200	0%	Light transmission aggregometry, HPR: AGGmax ≥34%	HPR patients received clopidogrel (600 mg loading dose and 75 mg maintenance dose)	HPR patients received clopidogrel (600 mg loading dose and 150 mg maintenance dose)	12	- MACE was significantly lower in the high-dose arm (high-dose 3.1% vs. low dose 24.6%, *p* = 0.01) - One major bleeding event occurred in the high-dose arm.
EFFICIENT (50)	192	0%	VerifyNow, HPR: percent inhibition <40%	Group 1: No HPR on clopidogrel 75 mg Group 2: HPR on clopidogrel 75 mg Group 3: HPR on clopidogrel 150 mg		6	- MACE was significantly higher in group 2 (17%) when compared to group 1 (5.1%, *p* = 0.019) and group 3 (4.3%, *p* = 0.045) - Minor bleeding rate was similar between all groups (group 1−4.1%, group 2−2.1%, and group 3−6.4%) - Only one major bleeding event occurred in group 3
Hazarbasanov (51)	192	56.8%	MAP, HPR: ADPTest aggregation value >46 units.	All patients received clopidogrel (300–600 mg loading dose and 75 mg maintenance)	Patients with HPR received clopidogrel 300–600 mg loading dose and additional 600 mg loading dose and 150 mg maintenance dose for 1 month.	6	- MACE was significantly lower in the PFT-guided arm (PFT-guided 0% vs. control 5.3%, *p* = 0.03). - Only one major bleeding event occurred in the PFT-guided arm, and zero events in the control arm
TRIGGER-PCI (52)	423	0%	VerifyNow, HPR: >208 PRU	Patients with HPR received clopidogrel 75 mg	Patients with HPR received prasugrel 10 mg	6	- MACE was similar between the two arms (Prasugrel 1.0% vs. clopidogrel 2.9%, *p* = 0.404) - Non-CABG all bleeding was similar between the two arms (Prasugrel 2.9% vs. clopidogrel 1.9%, *p* = 0.516)
ARCTIC (53)	2,440	27%	VerifyNow and Light transmitter aggregometry, HPR: ≥235 PRU or platelets inhibition >15%. LPR: platelet inhibition >90%.	Patients received clopidogrel or prasugrel per the clinician's discretion	Patients underwent PFT prior to PCI, and 2–4 weeks after. Clopidogrel or prasugrel were given and their doses were changed according to the PFT results	12	- MACE was similar between the two arms (PFT-guided 34.6% vs. control 31.1%, *p* = 0.10). - Major or minor bleeding rate was statistically lower in the PFT-guided arm without statistical significance (PFT-guided 3.1% vs. control 4.5%, *p* = 0.08).
ANTARCTIC (54)	877	100%	VerifyNow, HPR: ≥208 PRU. LPR: ≤ 85 PRU.	All patients received prasugrel 5 mg	Patients received prasugrel 5 mg and underwent PFTs 14 days after randomization, and 14 days after that. Dose adjustment were made depending on the PFT results	12	- MACE was similar between the two arms (PFT-guided 10% vs. control 9%, *p* = 0.80). - Major or minor bleeding rate was similar between the two arms (PFT-guided 38% vs. control 39%, *p* = 0.87).
Zhu et al. (55)	305	100%	Light transmitter aggregometry, HPR: platelets inhibition <10%.	All patients received clopidogrel (loading 600 mg, and 75 mg maintenance)	Patents with HPR received cilostazol 100 mg twice daily for 6 months in addition to clopidogrel (600 mg loading, and 75 mg maintenance), while patients with no HPR only received clopidogrel	12	- MACE was similar between the two arms (PFT-guided 9.7% vs. control 14.6%, *p* = 0.197). - No major bleeding events occurred in either of the groups.
TROPICAL ACS (56)	2,610	100%	MAP, HPR: ADPTest aggregation value >46 units.	All patients received prasugrel	De-escalation arm: Patients were started on 1-week prasugrel followed by 1-week clopidogrel, then based on the testing results. Patients with HPR were switched back to prasugrel while patients with no-HPR were continued on clopidogrel	12	- MACE was similar between the two arms (de-escalation 3% vs. control 3%, *p*_non−inferiority_= 0.0115). - Major bleeding rate was similar between the two arms (de-escalation 5% vs. control 6%, *p* = 0.23).
CREATIVE (57)	1,078	Not available	TEG, HPR: MA_ADP_ >47 mm plus an ADP-induced platelet inhibition rate <50%.	Standard arm: HPR patients on clopidogrel 75 mg Double arm: HPR patients on clopidogrel 150 mg Triple arm: HPR patients on clopidogrel 75 mg and cilostazol 100 mg twice daily		18	- MACE was significantly lower in the triple arm when compared to the standard arm (triple 8.5% vs. standard 14.4%, *p*-value not available) - MACE was similar between standard arm and double arm (double 10.6% vs. standard 14.4%, *p*-value not available) - Major bleeding rate was similar across all groups (standard 1.93%, double 3.34%, triple 2.53%)
PATROL (58)	1,353	100%	VASP, HPR: VASP >50%	-No HPR: No HPR patients on standard dose clopidogrel - HPR-clopidogrel: HPR patients on standard dose clopidogrel - HPR-ticagrelor: HPR patients on ticagrelor		12	- MACE was significantly higher in HPR-clopidogrel arm (19.49%) when compared to No-HPR (10.20%, p <0.05), and HPR-ticagrelor (8.57%, p <0.05). Major bleeding was similar across all groups (No-HPR 4.22%, HPR-clopidogrel 1.54%, HPR-ticagrelor 4.76%)
Zheng (59)	2,237	0%	Platelet aggregation detection device (PL-12), HPR: maximum aggregation rate >55%	All patients received clopidogrel 75 mg daily	Patients with HPR received ticagrelor 90 mg twice daily, and patients without HPR received clopidogrel 75 mg daily.	6	- MACE was significantly lower in the PFT-guided arm (PFT-guided 2.3% vs. control 4.7%, *p* = 0.003). - Major bleeding rate was similar between the two arms (PFT-guided 2.1% vs. control 1.6%, *p* = 0.360).
**Non-randomized studies**							
MADONNA (60)	798	37%	MAP, HPR: ADPTest aggregation value >50 units.	HPR patients on standard clopidogrel dose	HPR patients on repeated clopidogrel loading doses or prasugrel	1	- Stent thrombosis rate was lower in the guided arm (standard 1.9% vs. guided 0.2%, *p* = 0.027) - Major bleeding rate was similar between the two arms (standard 0.3% vs. guided 1%, *p* = 0.186)
ISAR-HPR (61)	999	50.3%	MAP, HPR: ≥468 aggregation units x minutes	HPR patients on standard clopidogrel	HPR patients with either reloading with clopidogrel, switching to prasugrel or kept on standard clopidogrel dose with monitoring of platelet function	1	- The rate of death or stent thrombosis was significantly lower in the guided arm (guided 1.2% vs. control 3.7%, *p* = 0.009) - Major bleeding rate was similar between the two arms (guided 1.9% vs. control 0.7%, *p* = 0.10)
PECS (62)	741	100%	MAP, HPR: ADPTest aggregation value >46 units.	Standard arm: No HPR patients on clopidogrel 75 mg Double arm: HPR patients on clopidogrel 150 mg Prasugrel arm: HPR patients on prasugrel		12	- MACE and major bleeding were significantly higher in the double arm when compared to standard arm - MACE: double 22.66% vs. standard 10.92%, *p* < 0.0001 - Major bleeding: double 9.38% vs. standard 4.79%, *p* = 0.04 - MACE and major bleeding were similar between standard arm and prasugrel arm - MACE: prasugrel 9.89%, *p* = 0.76 - Major bleeding: prasugrel 2.2%, *p* = 0.28.
PASTOR (63)	175	100%	VerifyNow, HPR: ≥235 PRU.	Patients without HPR on clopidogrel	Patients with HPR switched to prasugrel or ticagrelor	24	- MACE was similar between the two arms (No-HPR clopidogrel 7.0% vs. HPR-switched 8.7%, *p* = 0.70)
TOPIC-VASP (64)	645	100%	VASP, LPR: VASP ≤ 20%	All patients received 1 month of a non-clopidogrel P2Y12 after stent placement, then divided into 4 groups 1. Unchanged-LPR: patients on non-clopidogrel P2Y12 inhibitor with LPR 2. Unchanged-No LPR: patients on non-clopidogrel P2Y12 inhibitor without LPR 3. Switched-LPR: patients switched to clopidogrel and have LPR 4. Switched-No LPR: patients switched to clopidogrel and don't have LPR		12	Ischemic events: - Unchanged-LPR (14.9%), Unchanged-No LPR (8.3%), switched-LPR (6.6%), and switched no-LPR (11.7%) - Ischemic events were significantly lower in the unchanged no-LPR when compared to unchanged LPR (*p* = 0.04) - Ischemic events were significantly lower in the switched LPR when compared to the unchanged LPR (*p* = 0.02) - Major bleeding: - Unchanged-LPR (18.2%), unchanged-No LPR (11.8%), switched-LPR (5.3%), and switched no-LPR (2.9%) - Major bleeding rate was significantly lower in the switched-LPR when compared to unchanged LPR (*p* < 0.01) - Major bleeding rate was significantly lower in the switched no-LPR when compared to the unchanged no-LPR (*p* < 0.01)
Komocsi et al. (65)	2,104	100%	MAP, HPR: ADPTest aggregation value >46 units	Control arm, no PFT testing	PFT-guided: patients with HPR were recommended to switch to prasugrel	12	- MACE was significantly lower in the guided arm (guided 9.2% vs. control 12%, *p* < 0.05) - Rate of blood transfusion was similar between the two arms (guided 7.0% vs. control 6.4%, *p* > 0.05)
Dang et al. (66)	511	100%	Photo-turbidimetry	Control arm in which patients received DAPT for 12 months	PFT-guided arm where patients received adjusted doses and shortened DAPT duration according to their PFT results.	12	- MACE was similar between the two arms (guided 4.89% vs. control 6.07%, *p* = 0.41) - Major bleeding rate was lower in the guided-arm (guided 1.96% vs. control 4.5%, *p* = 0.037)

The ARCTIC study was a multi-center RCT that included a total of 2,440 undergoing PCI (53). VerifyNow was used for PFT measurements; HPR was defined as PRU ≥ 235 or platelets inhibition ≤ 15%, and low platelet reactivity (LPR) was defined as platelet inhibition >90%. Patients were randomized to the control arm where patients received clopidogrel or prasugrel per the clinician's discretion without PFT or PFT-guided arm where they undergo PFT prior to PCI, and 2 to 4 weeks after, and the P2Y12 agent (clopidogrel or prasugrel) and its dose were changed according to the PFT results. At 1 year, MACE was similar between the two arms (PFT-guided 34.6% vs. control 31.1%, *p* = 0.10). Major or minor bleeding rate was statistically lower in the PFT-guided arm without statistical significance (PFT-guided 3.1% vs. control 4.5%, *p* = 0.08) (53, 54). The ANTARCTIC study was an open-label, superiority RCT that included 877 patients with ACS undergoing PCI (54). VerifyNow was used for PFT measurements, and HPR was defined as PRU ≥ 208 while LPR was defined as PRU ≤ 85. Patients were randomized to the control arm where patients received prasugrel 5 mg without PFTs or PFT-guided arm where patients received prasugrel 5 mg and underwent PFTs 14 days after randomization, and 14 days after that. Dose adjustments were made depending on the PFT results. The primary end point of MACE and major bleeding was similar between the two arms (PFT-guided 28% vs. control 28%, *p* = 0.98) (54).

The TROPICAL ACS (67) study is a randomized, open-label, multi-center trial that was performed at 33 sites in Europe. A total of 2,610 patients with ACS who underwent a successful PCI were enrolled and followed up for 12 months. MAP was used for PFT measurements, and all patients underwent PFT. HPR was defined as adenosine diphosphate test (ADPTest) aggregation value >46 units. Patients were randomly assigned to two groups; a control arm where all patients received prasugrel irrespective of their PFT results, and a de-escalation arm. The de-escalation arm was based on PFT. Patients in this group were started on 1-week prasugrel followed by 1-week clopidogrel, then based on the testing results. Patients with HPR were switched back to prasugrel while patients with no-HPR were continued on clopidogrel. At 1 year follow up, the rate of MACE was similar between the two arms (de-escalation 3% vs. control 3%, *p*_non−inferiority_ = 0.0115) as well as the rate of major bleeding (de-escalation 5% vs. control 6%, *p* = 0.23) (67).

A pre-specified sub-study of the TROPICAL ACS study was conducted and aimed to assess clinical outcomes according to HPR and LPR in the included patients (56). HPR was defined as ADPTest > 46 units and LPR was defined as ADPTest <18 units. Twenty-seven percent of the control arm treated with prasugrel had LPR, while 15% had HPR. In the de-escalation arm, 11% had LPR, and were on clopidogrel while 40% had HPR and were on prasugrel. At 1 year, MACE and major bleeding were similar between patients without HPR on prasugrel (2.3%) when compared to patients without HPR on clopidogrel (2.4%, *p* = 0.86). Ischemic events were significantly higher in the control patients with HPR on prasugrel (4.8%) when compared to control patients without HPR on prasugrel (2.2%, *p* = 0.049). The risk of major and minor bleeding was significantly higher in the LPR patients (7.4%) when compared to patients with no-LPR (4.3%, *p* = 0.005) irrespective of the anti-platelet agent used (56).

### Evidence of Genotype-Guided P2Y12 Inhibitors

#### Non-randomized Studies

Over the last decade, multiple non-randomized studies have investigated the clinical benefit of a genotype-guided protocol for P2Y12 inhibitor selection (Table 3). In a prespecified *post-hoc* analysis, Wallentin et al. examined DNA samples from 10,285 participants from the PLATO trial. Patients were divided into four arms; patients with LOF alleles (CYP2C19^*^2 or ^*^3) treated with clopidogrel, patients with LOF alleles treated with ticagrelor, patients without LOF alleles treated with clopidogrel, and patients without LOF alleles treated with ticagrelor. At 12 months, the risk of MACE was lower in patients with LOF on ticagrelor (8.6%) when compared to patients with LOF on clopidogrel (11.2%, *p* = 0.03) while the risk of major bleeding was similar between the two arms (LOF-ticagrelor 11.8% vs. LOF-clopidogrel 11.3%, *p* = 0.77). In the non-LOF arms, the risk of MACE and major bleeding were similar between patients with no LOF on ticagrelor and patients with no LOF on clopidogrel (80).

**Table 3:** Summary of nonrandomized studied that compared clinical outcomes based on genotype-guided P2Y12 inhibitors.

	**Population**	**Proportion of patients with ACS**	**CYP2C19 alleles**	**Control arm**	**Experimental arm**	**Follow up duration in months**	**Results**
Classens (68) *post-hoc* analysis of POPular Genetics	2,429	100% STEMI	CYP2C19 *2, *3, *17	The study ran 2 separate analysis: - The first analysis: Carriers of CYP2C19*17 on clopidogrel vs. NM on clopidogrel - The second analysis: non-LOF carriers on clopidogrel vs. patients on ticagrelor/prasugrel irrespective of their allele		12	In the first analysis: - The composite thrombotic outcome of CV death, MI, stroke, and ST was similar between the two groups (CYP2C19*17 – 3.8% vs. NM-−3.8%, *p* = 0.90). - The combined major and minor bleeding outcome was also similar between the two groups (CYP2C19*17 – 9.3% vs. NM – 11.2%, *p* = 0.21). In the second analysis: -The composite thrombotic outcome of CV death, MI, stroke, and ST was similar between the two groups (non-LOF on clopidogrel 3.4% vs. ticagrelor/prasugrel 2.5%, *p* = 0.62). -The combined major and minor bleeding outcome was significantly lower in the clopidogrel arm (non-LOF on clopidogrel 9.9% vs. ticagrelor/prasugrel 11.7%, *p* = 0.03). - Sensitivity analysis was performed to compare outcomes in all non-LOF patients: -The composite thrombotic outcome was similar between non-LOF on clopidogrel (3.4%) compared to non-LOF on ticagrelor/prasugrel (2.9%, *p* = 0.72) -The combined major and minor bleeding outcome was significantly lower in the non-LOF on clopidogrel (9.9%) compared to non-LOF on ticagrelor/prasugrel (14.1%, *p* < 0.001)
PHARM-ACS (69)	1,361	100%–STEMI	CYP2C19 *2, *3, *17	1-Non-LOF treated with clopidogrel 2- Non-LOF treated with ticagrelor 3- LOF treated with clopidogrel 4- LOF treated with ticagrelor		15.6	- MACE was significantly higher in LOF-clopidogrel compared to LOF-ticagrelor (7.8% vs. 4.0%, *p* = 0.029, respectively). - MACE was similar between LOF-ticagrelor, non-LOF ticagrelor, and non-LOF clopidogrel. **LOF-ticagrelor (4.0%) vs. non-LOF clopidogrel (5.8%, *p* = 0.272). ** LOF-ticagrelor (4.0%) vs. non-LOF ticagrelor (4.3%, *p* = 0.846). **Non-LOF ticagrelor (4.3%) vs. non-LOF clopidogrel arms (5.8%, *p* = 0.99).
GIANT (70)	1,118	100%	CYP2C19 *2, *3, *4, *5, *6, *17	1-Class 1: NM, RM, and UM. 2- Class 2: PM, and IM treated with prasugrel or clopidogrel 150 mg. 3- Class 3: PM and IM with no treatment adjustment.		12	- The composite outcome of death, MI, and stent thrombosis was similar between class 1 (3.04%) and class 2 (3.31%) but significantly higher in class 3 (15.6%). - MACE and major bleeding events were similar between class 1 and class 2: - MACE: class 1- 8.6% vs. class 2- 9.9% - Bleeding: class 1- 1.8% vs. class2- 2.2%
Martin (71)	928	54.3%	CYP2C19 *2, *3, *17	1-IM/PM- on clopidogrel 2- IM/PM-on prasugrel or ticagrelor 3- UM/RM/NM-on clopidogrel 4- UM/RM/NM-on prasugrel or ticagrelor 5- IM/PM-initially on clopidogrel escalated to prasugrel or ticagrelor (escalation arm) 6- UM/RM/NM-initially on prasugrel or ticagrelor de-escalated to clopidogrel (de-escalation arm)		9.2	- MACE was significantly higher in IM/PM- on clopidogrel arm (26.4%) when compared to escalation arm (6.7%, *p* < 0.001) - MACE was similar between UM/RM/NM-clopidogrel arm (13.9%) when compared to escalation (6.7%, *p* = 0.038) - MACE was similar between de-escalation arm (10.1%) when compared to IM/PM-prasugrel or ticagrelor (8.9%) and UM/RM/NM-prasugrel or ticagrelor - (10.7%, *p* = 0.878)
Lee (22)	3,342	69%	CYP2C19 *2, *3, *17	1-IM/PM- on clopidogrel 2- NM- on clopidogrel 3- UM/RM- on clopidogrel 4- On prasugrel or ticagrelor irrespective of genotype		6.3	- MACE was significantly higher in the IM/PM-on clopidogrel arm (15.1%) when compared to prasugrel or ticagrelor irrespective of genotype arm (9.0%, *p* = 0.008). - MACE was similar between prasugrel or ticagrelor irrespective of genotype arm (9.0%) when compared to NM-on clopidogrel arm (10.4%, *p* = 0.993) and UM/RM- on clopidogrel (9.9%, *p* = 0.734).
Tan (72)	677	80.9%	CYP2C19 *2, *3	No genotype testing, patient received clopidogrel	Genotype guided: IM and PM received ticagrelor NM received clopidogrel	18	- MACE was significantly lower in the guided arm (guided 4.3% vs. control 8.4%, *p* = 0.027) -Major and minor bleeding risks were similar between the two arms (guided 2.2% vs. control 3.2%, *p* = 0.385)
IGNITE (73)	1,815	67%	CYP2C19 *2, *3	1-LOF-clopidogrel (clopidogrel 75 mg) 2-LOF-alternative (prasugrel, ticagrelor, or high dose clopidogrel of 150 mg daily) 3-Non-LOF (clopidogrel 75 mg daily, or prasugrel or ticagrelor)			- MACE was significantly higher in the LOF-clopidogrel (7.96%) when compared to LOF-alternative (4.62%). - MACE was similar between LOF-alternative (4.62) and non-LOF groups (5.95%).
Ozawa (74)	65	100%	CYP2C19 *2, *3	Control arm where patients received clopidogrel	IM and PM received prasugrel at least for 2 weeks		- MACE was significantly lower in the genotype guided arm (genotype 4.2% vs. control 22%, *p* = 0.02) - Major bleeding ware was not statically different between the two arms (genotype 0% vs. control 3.1%, *p* = 0.17)
Lee (75)	1,193	53.8%	CYP2C19 *2, *3, *17	1-IM/PM- on clopidogrel 2- IM/PM-on prasugrel or ticagrelor 3- UM/RM/NM-on clopidogrel 4- UM/RM/NM-on prasugrel or ticagrelor		8.7	- MACE was significantly higher in the IM/PM- on clopidogrel arm (26.5%) when compared to IM/PM-on prasugrel or ticagrelor arm (7.9%, *p* < 0.001). - MACE was similar between IM/PM-on prasugrel or ticagrelor (7.9%) when compared to UM/RM/NM-on clopidogrel (13.1%, *p* = 0.075) and UM/RM/NM-on prasugrel or ticagrelor (9.7%, *p* = 0.601)
Chen (76)	212		CYP2C19 *2	1-IM/PM-on clopidogrel 75 mg 2- IM/PM-on clopidogrel 150 mg for 1 month then 75 mg 3- IM/PM-on clopidogrel and tongxinluo 4- IM/PM-on ticagrelor		12	- Total adverse cardiovascular events was significantly higher in the IM/PM-on clopidogrel 75 mg (30.4%) when compared to IM/PM-on clopidogrel 150 mg (8%, *p* < 0.01), IM/PM-on clopidogrel and tongxinluo (10%, *p* < 0.01), and IM/PM-on ticagrelor (5.2%, *p* < 0.01). - No severe bleeding events occurred but the rate of mild events was significantly higher in the IM/PM- on ticagrelor arm (19.2%) when compared to IM/PM-on clopidogrel 75 mg (2.1%, *p* < 0.01), IM/PM- on clopidogrel 150 mg (10%, *p* < 0.01), and IM/PM- on clopidogrel and tongxinluo (6.8%, *p* < 0.01).
Deiman (77)	89	0%	CYP2C19 *2, *3, *17	PM- on clopidogrel	PM-on prasugrel	18	- MACE was significantly higher in the PM- on clopidogrel when compared to PM- on prasugrel (31 vs. 5%, *p* = 0.003, respectively).
Sanchez-Ramos (78)	719	86%	CYP2C19 *2, *3, and ABCB1 SNP	No genotype testing—routine clinical practice	Genotype guided; Carriers of CYP2C19*2, *3, and/or ABCB1 received prasugrel or ticagrelor Non-carriers received clopidogrel	12	- MACE was significantly lower in the genotype-guided arm when compared to the control arm (10.1% vs. 14.1%, *p* = 0.037, respectively). - TIMI major bleeding rate was similar between the two arms (Genotype guided 1.9% vs. 2.5% in non-genotype guided, *p* = 0.59) -TIMI minor bleeding rate was similar between the two arms (Genotype guided 2.2 vs. 2.2% in non-genotype guided, *p* = 0.97)
Shen (79)	628	Not reported	CYP2C19 *2, *3	No genotype testing—Clopidogrel 75 mg daily	NM: *1,*1- clopidogrel 75 mg daily IM: *1,*2 or *1,*3 - clopidogrel 150 mg daily PM: *2,*2 or *2,*3 or *3,*3 - ticagrelor 90 mg twice daily	12	- MACE was significantly lower in the genotype-guided arm at 1, 6, and 12 months. - 1 month (Genotype 1.3 vs. 5.6%, *p* = 0.0003) - 6 months (Genotype 3.2 vs. 7.8%, *p* = 0.012) - 12 months (Genotype 4.2 vs. 9.4%, *p* = 0.01). - Bleeding events were similar between the two groups at 1, 6, and 12 months. - 1 month (Genotype 4.9 vs. 3.4%, *p* = 0.38) - 6 months (Genotype 6.8 vs. 5.0%, *p* = 0.34) - 12 months (Genotype 8.1 vs. 6.0%, *p* = 0.29)
Wallentin (80), *post-hoc* analysis of PLATO	10,285	100%	CYP2C19 *2, *4, *4, *5, *6, *7, *8, *17 ABCB1	1-Non-LOF treated with clopidogrel 2- Non-LOF treated with ticagrelor 3- LOF treated with clopidogrel 4- LOF treated with ticagrelor		12	- The composite outcome of cardiovascular death, MI, or stroke was lower in the LOF on ticagrelor when compared to LOF on clopidogrel while major bleeding rate was equal between the two arms - MACE: LOF ticagrelor (8.3%) vs. LOF clopidogrel (10.7%, *p* = 0.03) - Major bleeding: LOF ticagrelor (11.8%) vs. LOF clopidogrel (11.3%, *p* = 0.77) - The composite outcome of cardiovascular death, MI, or stroke and major bleeding rate were similar between non-LOF ticagrelor vs. non-LOF clopidogrel - MACE: non-LOF ticagrelor (8.3%) vs. non-LOF clopidogrel (9.7%, *p* = 0.06) - Major bleeding: non-LOF ticagrelor (9.3%) vs. LOF clopidogrel (9.7%, *p* = 0.61)
Mega (19), *post-hoc* analysis of TRITON-TIMI 38	1,477	100% (71% STEMI, 29% NSTEMI)	CYP2C19, CYP2C9, CYP2B6, CYP3A5, CYP3A4, and CYP1A2	Non-carriers of LOF alleles on clopidogrel	Carriers of LOF alleles on clopidogrel	15	- Composite outcomes of cardiovascular death, MI, or stroke was significantly higher in the carriers of LOF alleles (Carriers 12.1% vs. Non-carriers 8%, *p* = 0.01) - Major or minor bleeding risk was similar between the two groups (Carriers 3.2% vs. Non-carriers 3.06%)

The IGNITE network was a multisite investigation of genotype-guided P2Y12 inhibitors at seven different sites across the United States (73). A total of 1,815 patients were included and divided into 3 groups; patients with 1–2 LOF alleles who received clopidogrel 75 mg daily (LOF-clopidogrel group), patients with 1–2 LOF alleles who received alternative therapy (prasugrel, ticagrelor, or high dose clopidogrel of 150 mg daily) (LOF-alternative group), and patients without LOF alleles who received clopidogrel 75 mg daily, or prasugrel or ticagrelor (Non-LOF group). At 12 months, MACE was significantly higher in the LOF-clopidogrel group when compared to LOF-alternative group but similar between the LOF-alternative and non-LOF groups. Within the non-LOF group, MACE was also similar between non-LOF treated with clopidogrel vs. non-LOF treated with prasugrel or ticagrelor. Sixty-seven percent of the total population presented with ACS. Subgroup analysis in patients presented with ACS showed similar results as the total population (73).

The GIANT study was a multisite, prospective observational study conducted in France (70). A total of 1,118 patients presenting with ST-elevation myocardial infarction (STEMI) were included. The study protocol recommended PM to be treated with prasugrel, IM to be treated with prasugrel or clopidogrel 150 mg daily, NM, RM, and UM treatment to be left to the clinician's discretion. Patient's class was defined according to their genotype; NM, RM, and UM were defined as class 1, IM and PM who were treated per the protocol were defined as class 2, and IM and PM who weren't treated per the protocol were defined as class 3. At 12 months, MACE was similar between class 1 and class 2 (8.6 vs. 9.9%; *p* = 0.45, respectively), as well as major bleeding events (1.8 vs. 2.2%; *p* = 0.45, respectively). Patients in class 3 had significantly higher rates of combined death, MI, and stent thrombosis when compared to class 1 or class 2 (3.04% in class 1 vs. 3.31% in class 2 vs. 15.6% in class 3; *p* < 0.05 vs. class 1 and class 2) (70).

The PHARM-ACS study; a single center observational study conducted in China (69). A total of 1,361 patients were included and divided into four groups according to their LOF alleles and P2Y12 inhibitor use; Patients with LOF-alleles treated with clopidogrel (38.5%), patients with LOF-alleles treated with ticagrelor (22.2%), patients without LOF-alleles treated with clopidogrel (29.2%), and patients without LOF-alleles treated with ticagrelor (10.1%). MACE was significantly higher in the LOF-clopidogrel group when compared to the LOF-ticagrelor arm (7.8 vs. 4.0%, *p* = 0.029, respectively). Additionally, MACE was similar between the LOF-ticagrelor, non-LOF ticagrelor, and non-LOF clopidogrel arms (4.0, 4.3, and 5.8%, respectively).

#### Randomized Clinical Trials

As detailed above, multiple observational studies have shown clinical benefit of a genotype-guided antiplatelet therapy. To examine the impact of genetics on clinical outcomes several RCTs were conducted to explore the efficacy of a genotype-guided regimen (Table 4).

**Table 4:** Summary of randomized control trials that compared clinical outcomes based on genotype-guided P2Y12 inhibitors.

	**Population**	**Patients with ACS**	**CYP2C19 alleles**	**Control arm**	**Experimental arm**	**Follow up duration in months**	**Results**
IAC-PCI (81)	600	100%	CYP2C19 *2, *3	All received clopidogrel (load 300 mg, maintenance 75 mg)	NM—received clopidogre (load 300 mg, maintenance 75 mg) IM—received clopidogrel (load 600 mg, maintenance 150 mg) PM—received cilostazol (load 200 mg, and maintenance 100 mg twice daily) and also received clopidogrel (load 600 mg, maintenance 150 mg)	6	- MACE was significantly lower in the guided arm (guided 2.66% vs. control 9.03%, *p* = 0.001) - Bleeding events were similar between the two arms (guided 1.33% vs. control 3.68%, *p* = 0.001)
Xiong (82)	224	100%	CYP2C19 *2	PM treated with clopidogrel 150 mg once daily	PM treated with Ticagrelor 90 mg twice daily	1	- Platelet reactivity inhibition was significantly different between the two groups. Ticagrelor was a faster onset of action in the platelet inhibition and an ~2-fold higher potency than clopidogrel - No MACE or major bleeding events occurred in both groups during the study period - Minor bleeding was lower in the ticagrelor arm (ticagrelor 7.1% vs. clopidogrel 20.5%, p=0.001)
Ogawa (83), *post-hoc* analysis of PRASFIT-ACS	773	100%	CYP2C19 *1*2*3	Patients received clopidogrel (load 300 mg, maintenance 75 mg) divided into 3 subgroups NM (35.2%) IM (44.6%) PM (20.1%)	Patients received adjusted prasugrel dose (load 20 mg, maintenance 3.75 mg) divided into 3 subgroups NM (39.2%) IM (41.0%) PM (19.7%)	12	- In IM/PM patients, MACE was similar between the two arms (prasugrel 10.5% vs. clopidogrel 12.5%), *p*-value not available. Similar findings were observed in the total populations, and in NM subgroup. - In IM/PM patients, all bleeding events were significantly higher in the prasugrel arm (prasugrel 50.2% vs. clopidogrel 31.9%), *p*-value not available. Similar findings were observed in the total populations. However, the risk of major bleeding was similar between all study groups.
Dong (84)	166	100%	CYP2C19*1*2*3	Patients on clopidogrel divided into 3 subgroups NM IM PM	Patients on ticagrelor divided into 3 subgroups NM IM PM		- Inhibition of platelet aggregation was significantly higher in the total and all subgroups of the ticagrelor arm when compared to the total and all subgroups of the clopidogrel arm (*p* < 0.05) - MACE was numerically lower in the total ticagrelor arm (29.6%) when compared to the total clopidogrel arm but did not reach statistical significance (44.1%, *p* > 0.05) - MACE was significantly lower in PM of the ticagrelor arm when compared to PM of the clopidogrel arm (*p* > 0.05) - MACE was significantly lower in NM, and IM of the clopidogrel arm when compared to PM of the clopidogrel arm (*p* > 0.05)
PHARMCLO (85)	888	100% (25.5% STEMI 70.5% NSTEMI)	CYP2C19 *2*17 ABCB1	Received standard of care therapy (clopidogrel 50.7%, prasugrel 8.4%, ticagrelor 32.7%)	Genetic testing results integrated into an algorithm to guide therapy (clopidogrel 43.3%, prasugrel 7.6%, ticagrelor 42.6%) (pharmacogenomic arm)	12	- Composite of cardiovascular death and the first occurrence of non-fatal myocardial infarction, non-fatal stroke, and major bleeding was significantly lower in the pharmacogenomic arm (15.9%) compared to control arm (25.9%; p <0.001) - In the subgroup of patients on clopidogrel, the composite outcome was significantly lower in the pharmacogenomic arm (24.7%) compared to control arm (35.4%; *p* = 0.03)
POPular Genetics (86)	1,242	100% STEMI	CYP2C19 *2*3	No genetic testing, and all patients received ticagrelor or prasugrel	Carriers of LOF alleles received ticagrelor or prasugrel while non-carriers received clopidogrel	12	- The composite outcome of all-cause death, MI, ST, stroke, or major bleeding was non-inferior in the genotype arm (5.9) when compared to standard therapy (5.1%, *P*_non−inferiority_ <0.001). - Major and minor bleeding rates were significantly lower in the genotype-guided group (9.8) compared to standard therapy (12.5%; *P* = 0.04)
Tuteja (87)	504	50%	CYP2C19*2 *3 *17	No genetic testing (79% clopidogrel, 21% prasugrel/ticagrelor)	Underwent genetic testing but choice left to the physician. NM/RM (clopidogrel 78%, ticagrelor/prasugrel 22%) IM/PM (clopidogrel 47%, ticagrelor/prasugrel 53%)	16.4	- The composite outcome of cardiovascular death, MI, stroke, urgent revascularization, and ST was similar in the genotype arm (13.7%) when compared to the control arm (10.2%, *p* = 0.32). Subgroup analysis in ACS patients was also not significant. - Major bleeding events were also similar between the two groups (control 5.1% vs. genotype 5.2%, *p* > 0.05) - After study completion, genetic analysis was performed in all patients, and *post-hoc* analysis was conducted: - The composite MACE and major bleeding outcome was significantly higher in LOF-carriers on clopidogrel (21.2%) when compared to all no-LOF (12.9%, *p* = 0.03) - The composite MACE and major bleeding outcome was numerically higher in LOF-carriers on ticagrelor/prasugrel (19.2%) when compared to all no-LOF but did not reach statistical significance (12.9%, *p* = 0.44)
Franchi (88)	781	100%	CYP2C19*1*2*3*17	PM/IM on prasugrel	PM/IM on ticagrelor	1	- No ischemic or major bleeding events were observed in either group. - In the prasugrel arm, one patient had a stroke vs. none in the ticagrelor arm - Pharmacodynamic assessment was performed and P2Y12 reaction unit was markedly reduced to a similar extent between the arms throughout the follow up period (*p* = 0.519).
TAILOR PCI (89)	5,302	82%	CYP2C19*2*3	No genetic testing, and all patients received clopidogrel	Genetic testing was performed, LOF-carriers received ticagrelor, and ono-LOF carriers received clopidogrel	12	- The primary composite outcome of CV death, MI, stroke, severe recurrent ischemia, ST was similar between the 2 arms (genotype 4.0% vs. control 5.9%, *p* = 0.06). - The combined major and minor bleeding rate was also similar between the 2 groups (genotype 1.9% vs. control 1.6%, *p* = 0.58). Genotype testing was performed after the completion of follow up in all patients and *post-hoc* analysis was conducted - The primary composite outcome was similar between non-LOF carriers in control arm (5.0%) when compared to experimental arm (4.7%, *p* = 0.73). - The combined major and minor bleeding outcome was similar between non-LOF carriers in control arm (1.0%) when compared to experimental arm (1.2%, *p* = 0.61).

The PHARMCLO trial included 888 patients with ACS, and randomized them into standard of care arm and pharmacogenomic arm (85). Patients in the pharmacogenomic arm underwent testing for CYP2C19^*^2, CYP2C19^*^17, and ABCB1 alleles. Clinical variables alone guided therapy in the control arm. In the pharmacogenomic arm, clinical variables, and results of the genetic testing were integrated in an algorithm to guide clinicians in their choice of antiplatelets therapy. The choice of P2Y12 agent was significantly different between the arms (*p* = 0.02); clopidogrel was used more frequently in the standard-of-care arm (50.7 vs. 43.3%), while ticagrelor was used more frequently in the pharmacogenomic arm (32.7 vs. 42.6%). Prasugrel was used similarly between the two arms (standard-of-care 8.4% vs. pharmacogenomic 7.6%). At 12 months, the composite outcome of cardiovascular death, first non-fatal MI, non-fatal stroke, and major bleeding was significantly lower in the pharmacogenomic arm (pharmacogenomic 15.9% vs. standard-of-care 25.9%, *p* < 0.001).

Claassens et al. conducted an open label randomized multicenter “POPular Genetics” RCT (86). The study included a total of 2,429 patients with STEMI who underwent PCI. Patients were randomized into two arms; control arm vs. genotype-guided arm. All patients in the control arm received ticagrelor or prasugrel. Patients in the genotype arm underwent genetic testing for LOF-alleles (CYP2C19^*^2 and CYP2C19^*^3). Carriers of LOF alleles received ticagrelor or prasugrel while non-carriers received clopidogrel. The composite outcome of death from any cause, MI, definite stent thrombosis, stroke, or major bleeding was similar between the two arms (genotype 5.9% vs. control 5.1%, *P*_non−inferiority_ <0.001), while the composite outcomes of major and minor bleeding was significantly lower in the genotype arm (genotype 9.8% vs. control 12.5%, *p* = 0.04).

A *post-hoc* analysis of the POPular Genetics study was recently published (68). In the original trial, blood samples were collected from both arms but only the genotype arm underwent genetic testing for CYP2C19^*^2 and CYP2C19^*^3. In this *post-hoc* analysis, genetic testing for CYP2C19^*^17 were performed on collected blood samples from all patients included in the trial (control and genotype arms). The study was designed to perform two separate analyses; the first analysis assessed the impact of CYP2C19^*^17 in patients treated with clopidogrel. Carriers of CYP2C19^*^17 treated with clopidogrel had similar rate of the composite thrombotic outcome of cardiovascular death, MI, ST and stroke (CYP2C19^*^17–3.8% vs. CYP2C19^*^1–3.8%, *p* = 0.90) as well as similar major and minor bleeding rates when compared to carriers of the wild type treated with clopidogrel (CYP2C19^*^17–9.3% vs. CYP2C19^*^1–11.2%, *p* = 0.21). The second analysis compared outcomes in patients without LOF alleles treated with clopidogrel vs. patients treated with ticagrelor or prasugrel irrespective of their alleles. No significant difference in the combined thrombotic outcome (cardiovascular death, MI, ST, and stroke) was found between the clopidogrel treated group vs. ticagrelor/prasugrel treated group (3.4 vs. 2.5%, *p* = 0.62, respectively). However, the combined major and minor bleeding outcome was significantly less frequent among clopidogrel treated group patients (clopidogrel 9.9 vs. 11.7% ticagrelor/prasugrel, *p* = 0.03).

The TAILOR-PCI study was conducted to determine the effect of genotype guided strategy in patients with ACS and stable CAD undergoing PCI (89). Patients included in the genotype arm underwent genetic testing for LOF alleles, and received ticagrelor if LOF alleles carriers, and clopidogrel is LOF alleles non-carriers. All patients in the control arm received clopidogrel and underwent genetic testing at the end of the follow up period. The primary analysis compared clinical outcomes between patients with LOF alleles who received ticagrelor in the genotype arm and patients with LOF alleles who received clopidogrel in the control arm. At 12 months of follow up, there was no significant difference in the composite outcome of cardiovascular death, MI, stroke, recurrent ischemia, and ST between the genotype arm compared to the control arm (genotype 4.0% vs. control 5.9%, *p* = 0.06) (89). The rate of combined major and minor bleeding was also similar between the 2 groups (genotype 1.9% vs. control 1.6%, *p* = 0.58). Even though this relatively large study randomized 5,302 patients, the trial's primary endpoint did not meet the predetermined level of statistical significance. These results should be interpreted in the context of the treatment effect (50% reduction in ischemic events) that the study was powered to detect based on the pre-specified analysis plan.

Parcha et al. performed a *post-hoc* Bayesian reanalysis of the TAILOR-PCI trial using informative and non-informative priors (90). Bayesian analysis was used to detect the posterior probability of reducing MACE using genotype-guided therapy after PCI. Using non-informative priors, the Bayesian analysis was conducted on the TAILOR-PCI trial without basing it on previous evidence. Using non-informative priors, the probability of risk ratio (RR) <1 for MACE was 94% using genotype-guided therapy. Informative priors were obtained from RCTs to add rigor for prior estimation and reduce the residual confounding in other study designs. The authors searched the literature for RCTs that assessed clinical outcomes of genotype guided therapy after PCI with a follow up period of at least 6 months, and included four RCTs; the ADAPT, POPular Genetics, IAC-PCI, and PHARMCLO trials. Using informative priors based on these trials, the probability of RR <1 for MACE was 99% using genotype-guided therapy indicating the benefit of such therapy in patients undergoing PCI (90).

## Meta-Analysis

Pereira et al. conducted a meta-analysis examining the effect of CYP2C19 genotyping in patients with CAD who underwent PCI treated with clopidogrel vs. ticagrelor or prasugrel (91). A total of seven RCTs and four non-RCTs were included. In the total populations, patients with LOF-alleles treated with ticagrelor or prasugrel had significantly lower ischemic events when compared to patients with LOF-alleles treated with clopidogrel (*p* = 0.035). A subgroup analysis in patients only from RCTs showed similar results regarding ischemic outcomes [ticagrelor/prasugrel 7.0% vs. clopidogrel 10.3% (RR: 0.70; 95% CI: 0.59–0.83)]. Patients with no LOF-alleles had similar ischemic outcomes regardless of antiplatelets agent used (RR 0.95; 95% CI 0.82–1.10). Bleeding outcomes were similar across all populations in the analysis (91).

Another meta-analysis included a total of 14 studies (11 RCTs, and 3 non-RCTs), and 20,743 patients aimed to assess the efficacy of guided antiplatelet therapy vs. conventional therapy in patients undergoing PCI (92). In this analysis, guided therapy was defined by genotype testing or PFT. Genetic testing was used in eight studies, while PFT was used in six. MACE was significantly lower in the guided therapy arm when compared to the standard therapy arm (RR 0.78, 95% CI 0.63–0.95) while the risk of any bleeding was similar between the two arms (RR 0.88, 95% CI 0.77–1.01). Subgroup analysis in RCTs only showed lower risk of MACE as well as lower any bleeding rate in the guided therapy arm compared to the standard therapy. Moreover, the authors conducted subgroup analysis according to the escalation vs. de-escalation protocols of the included studies (escalation in 10 studies, and de-escalation in 4 studies). In the escalation subgroup, MACE was significantly lower in the guided arm (RR 0.74, 95% CI 0.57–0.95), while the risk of any bleeding was similar between the two arms (RR 1.00, 95% CI 0.80–1.25). In the de-escalation subgroup, guided therapy results in a 10% risk reduction in MACE without statistical significance (RR 0.90, 95% CI 0.72–1.14), and resulted in a statistically lower risk of any bleeding (RR 0.81, 95% CI 0.68–0.96) when compared to standard therapy (92).

Galli et al. performed a network meta-analysis comparing guided antiplatelet therapy to different P2Y12 inhibitors (93). They included RCTs comparing different P2Y12 inhibitors in patients with ACS, as well as RCTs comparing guided therapy compared to conventional therapy. A total of 15 RCTs, 61,898 patients with ACS were included, and clopidogrel was used as the reference treatment group. In terms of MACE, guided therapy was associated with lower MACE when compared to clopidogrel therapy while prasugrel and ticagrelor were associated with similar risk of MACE when compared to clopidogrel. All bleeding risk was similar between clopidogrel and guided therapy but significantly higher in prasugrel and ticagrelor when compared to clopidogrel. Ranking of treatments were calculated according to p-scores. Guided therapy was the best treatment in terms of MACE, MI, all-cause death, stroke. Prasugrel was the best treatment for risk of stent thrombosis, ticagrelor was best treatment in terms of cardiovascular death, and clopidogrel was the best treatment in terms of bleeding risk. The authors concluded that guided therapy offers the most favorable balance between efficacy and safety when compared to routine selection of ticagrelor, or prasugrel (93).

## Cost Effectiveness

Multiple studies were conducted to evaluate the cost effectiveness of guided antiplatelet therapy in patients with CAD. Lala et al. conducted a cost-effective analysis of genotype-guided therapy in patients with ACS undergoing PCI. Patients were divided into three groups: patients on clopidogrel without genetic testing, patients on prasugrel without genetic testing, and patients with genotype-guided approach. Results were based on quality-adjusted life-years (QALY), and monetary cost in US dollars. Over 15 months, the genotype-guided arm had the highest QALY, and lowest cost when compared to the two arms. In terms of QALY, the genotype arm had a gain of 0.004 QALY compared to the clopidogrel arm, and 0.0005 QALY compared to the prasugrel arm. In addition, genotype arm had lower cost of $18 when compared to clopidogrel arm, and $899 when compared to prasugrel arm (94). Kim et al. performed cost-effectiveness analysis of genotype-guided, and PFTs-guided therapy in patients with ACS undergoing PCI (95). Patients were divided into multiple arms; patients placed universally on clopidogrel, patients universally on ticagrelor, patients underwent genetic testing and PM were placed on ticagrelor (conservative ticagrelor), patients underwent genetic testing and PM/IM were placed on ticagrelor (liberal ticagrelor), patients underwent PFTs and patients with HPR were placed on ticagrelor, patients underwent genetic testing and PFTs, and were placed on ticagrelor based on both results. The primary outcome was incremental cost effectiveness ratio (ICER) which was defined as the incremental cost divided by QALY gained over the lifetime horizon. Compared to universal clopidogrel, all the alternative therapies increased QALY as well as cost. In terms of ICER, PFT-guided, and liberal ticagrelor arms were the most cost-effective strategies ($12,119/QALY, and $29,412/QALY, respectively) (95).

Almukdad et al. performed a systematic review and identified 13 studies that investigated cost-effectiveness of genotype-guided therapy in patients with ACS undergoing PCI (96). They reported that six studies showed that genotype-guided therapy was cost-effective when compared to universal clopidogrel, while five studies showed that it was dominant. In addition, genotype-guided therapy was dominant when compared to universal prasugrel in five studies, to universal ticagrelor in one study, and to both in three studies. Out of the 13 included studies, only two studies showed that universal ticagrelor was cost-effective when compared to genotype-guided therapy. They concluded that genotype-guided therapy is cost-effective in patients with ACS undergoing PCI (96, 97). Most recently, Claassens et al. conducted a cost effectiveness analysis of genotype guided therapy based on the POPular Genetics trial (97). A base-case analysis was performed for a cohort of 1,000 patients showed that genotype guided therapy was dominant, and resulted in a gain of 8.98 QALYs, and cost saving of €725,550.69 when compared to universal ticagrelor or prasugrel (97).

### ABCD-GENE Score

**Figure 4:**
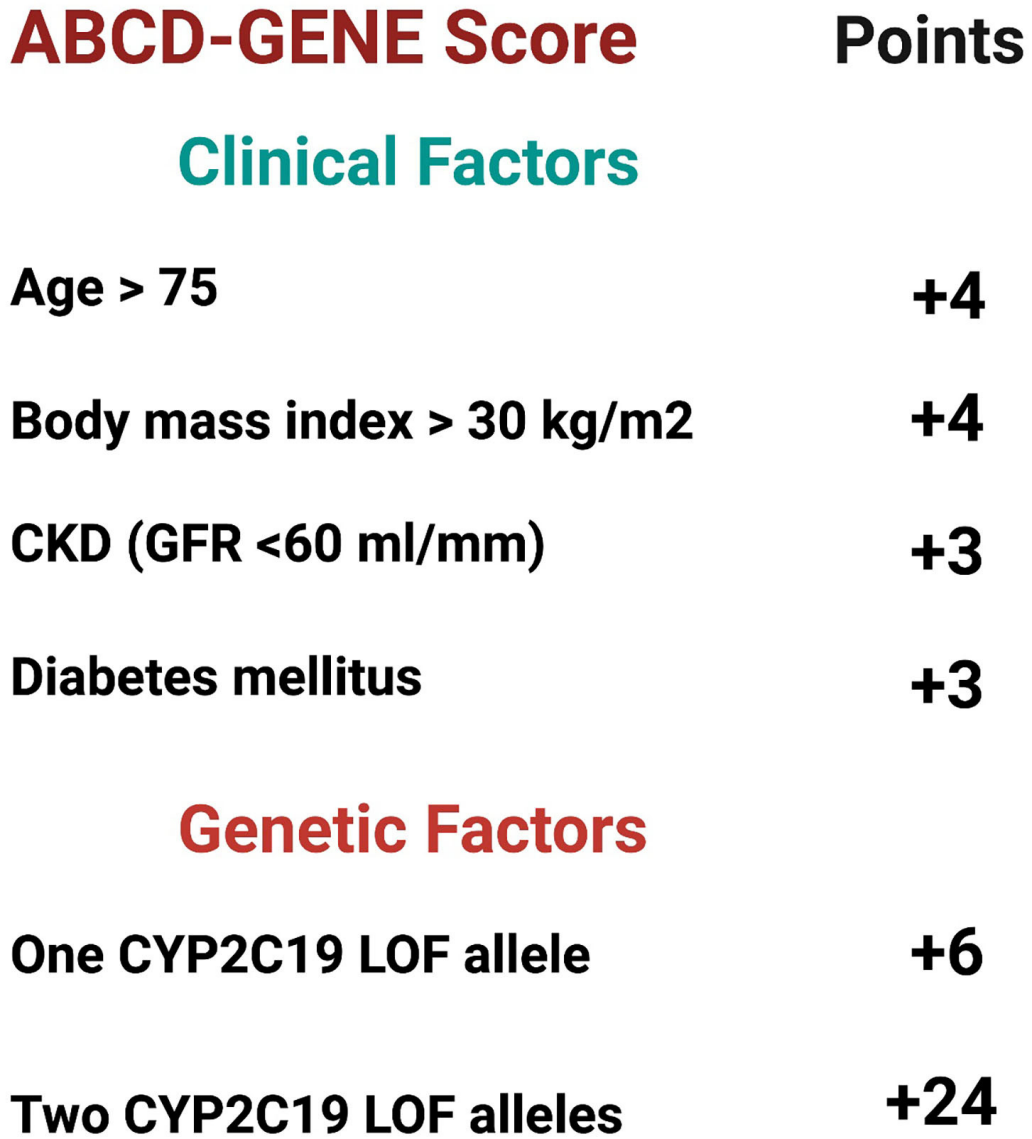
ABCD-Gene scoring system. The ABCD-GENE scoring system was developed to predict patients who are on P2Y12 inhibitors and at risk of high platelet reactivity (HPR). The scoring system was internally and externally validated. Patients with ABCD-Gene score ≥ 10 are at higher risk of all-cause death and major cardiovascular adverse events (MACE) at 1-year compared to those with a score <10. Created with BioRender.com (99). CKD, chronic kidney disease; CYP2C19, cytochrome P450 2C19; GFR, glomerular filtration rate; LOF, loss of function.

In addition to LOF-carrier status, multiple clinical factors can affect clopidogrel's responsiveness. These factors include diabetes mellitus (DM), age, and body mass index (BMI) (98). The ABCD-GENE (Age, BMI, chronic kidney disease, DM, and genotyping) score was developed to detect patients at risk of unresponsiveness to clopidogrel defined by HPR and worse clinical outcomes (99) (Figure 4). Angiolillo et al. derived the ABCD-GENE score based on the clinical characteristics of the patient with HPR in the GRAVITAS study. Clinical characteristics that were associated with HPR in the GRAVITAS population were externally validated using the POPULAR and FAST-MI registries. Thirty days HPR was the primary endpoint for external pharmacological validation and was validated using the POPULAR registry. One year all-cause death was the primary endpoint for external clinical validation, and was validated using the FAST-MI registry (99).

In multivariate analysis, age > 75 years, BMI > 30 kg/m^2^, chronic kidney disease (Glomerular filtration rate <60 ml/min), DM, and genotyping were independent predictors of HPR at 30-days. Based on the sum of the weighted predictors present in each case, bootstrap regression analysis was used to develop the score. To predict HPR at 30 days, a cutoff score of ≥10 resulted in the best sensitivity (47.9% in GRAVITAS population and 18.3% in the POPULAR population) and specificity (81.4% in GRAVITAS population and 92.6% in the POPULAR population). In regards to clinical validation, a cutoff score of ≥10 was associated with higher all-cause death at 1 year compared to a score <10 (17.3 vs. 7.5%, *p* = 0.002, respectively) (99).

Saito et al. (100) investigated the validation of the ABCD-GENE study in east Asian populations (101). A total of 184 patients were included from 4 different prospective studies. Patients with ABCD-GENE score of ≥10 were at increased risk of HPR on clopidogrel compared to patients with score of <10 (82 vs. 37%, *p* < 0.001).

## Current Guidelines Regarding Guided Therapy

The European Society of Cardiology (ESC) in 2018 has suggested a potential value for monitoring antiplatelet drugs on a prognostic level and for individualizing management plan (4). Based on a large detailed meta-analysis and meticulous review, a platelet reactivity assessment classified patients treated with PCI into two groups; a high on treatment platelet reactivity who are at higher risk of mortality and stent thrombosis, and a low on treatment reactivity who are at higher bleeding risk. Also, in patients who cannot complete 12 month of potent platelet inhibition, a guided de-escalation therapy of P2Y12 inhibitor therapy may be considered; with a downgrading from prasugrel or ticagrelor to clopidogrel (Class IIb, level of evidence: B). Although ESC consensus acknowledged the importance of genotype and platelet function studying in guiding the management plan to adjust P2Y12 inhibitor therapy, neither was recommended on a routine basis to tailor DAPT therapy following stenting in ACS-PCI patients. Although it was not recommended, genotype or PFT can still be considered to de-escalate therapy, evaluate compliance to treatment, and/or to prognosticate following PCI.

An expert consensus statement published in 2019 reported a lack of robust evidence, particularly from RCTs, to recommend the routine use of genotype or platelet function guided approach in patients undergoing PCI (28). However, in selected cases and individualized scenarios in patients with high bleeding or thrombotic risks, the use of PFT or genetic testing was proposed to help aid the clinical decision in choosing the most appropriate P2Y12 inhibitor. A DAPT escalation therapy may be used when thrombotic risk outweighs bleeding risk, and de-escalation approach when bleeding risk outweighs thrombotic risk.

ACC guidelines still do not recommend the routine use of PFT or genetic testing in clinical practice. The 2020 ESC guidelines for the management of non-ST elevation myocardial infarction still did not recommend routine platelet function testing or genotype testing in this set of patients with considering guided or unguided de-escalation in patients who are not suitable for 12 months of potent P2Y12 inhibitors (101).

## Conclusions

As reviewed above, numerous studies have examined the effectiveness and safety and efficacy of genotype-guided or platelet function-guided strategy to select antiplatelet therapy after PCI. However, a consensus has not been reached. Some studies have shown improved patient outcomes and lower MACE when using this personalized treatment strategy. A large national administrative claims database, the OptumLabs Data Warehouse, that includes longitudinal health data of more than 120 million individuals showed that Clopidogrel was prescribed in 61% of patients after PCI in 2018, ticagrelor in 31%, and prasugrel in 8%. Carriers of LOF alleles are common in the general population, 26% of Caucasians, 34% of African Americans, and up to 46% in Asians (102). Since genotype-guided or platelet-function guided treatments are not widely used, a large proportion of patients treated with clopidogrel after PCI are missing an opportunity for optimized treatment and improved outcomes.

Most contemporary studies showed that the use of newer generation stents was associated with much slower rates of thrombotic events (89, 103). In addition, at least two studies demonstrated favorable safety and effectiveness of short DAPT duration after PCI, as short as 1 month (104, 105). This raises a question of whether a personalized genotype-guided strategy still shows a benefit if the DAPT duration is short. The TAILOR-PCI trial showed that the potential effect of a precision medicine approach may be more important early after PCI, as suggested in their *post-hoc* analysis that demonstrated the potential benefit of genotype-guided oral P2Y12 inhibitor therapy in the first 3 months after PCI (89, 106).

To date, clopidogrel remains the preferred P2Y12 inhibitor used in combination with an anticoagulant in patients with atrial fibrillation requiring PCI, mainly because of concerns for increased bleeding with prasugrel or ticagrelor in combination with anticoagulation. Based on recent clinical trials, the use of clopidogrel as a single antiplatelet agent in combination with a direct oral anticoagulant (DOAC) was associated with decreased risk of bleeding and hospitalizations when compared to DAPT plus DOAC. However, the role of CYP2C19 genetic testing or PFT and their effect on thrombotic events in this scenario (anticoagulation plus clopidogrel without aspirin) remain unknown. Well-designed studies are needed to provide these answers in this patient population.

Racial and ethnic influence on antiplatelet therapy and response to genotype guided treatment strategy remain largely unknown. Approximately 35% of the African American population and up to 46% of the Asian population carry a CYP2C19 LOF allele (10), yet they are underrepresented in the contemporary genotype-guided antiplatelet trials. Asians constituted <3% of the patients included in the POPular Genetics trial and around 28% in the TAILOR-PCI trial while African Americans constituting <2% of patients enrolled in both trials (86, 89).

Several studies showed that implementing genotype guided antiplatelet therapy was a cost-effective approach compared to a non-guided use of antiplatelets after PCI (96). Although it is expected that the cost of ticagrelor and prasugrel will decrease when their generic forms become commercially available, it is also expected that the cost of genetic testing will be reduced in the future and may become more routinely available (107, 108). Therefore, it is still expected that a genotype guided strategy will remain cost effective, especially when the cost of increased bleeding with ticagrelor and prasugrel is taken into consideration.

Low dose ticagrelor (60 mg twice daily) is another potential alternative in patients with high bleeding risk or high ischemic risk who require prolonged duration of DAPT. The PEAGASUS-TIMI 54 trial investigated the benefit of long-term low dose ticagrelor 1 year after a patient's myocardial infarction. At 3 years, the reported decreased incidence of ischemic events in patients treated with aspirin and low dose ticagrelor when compared to aspirin and placebo. However, higher major and minor bleeding events occurred with the addition of low dose ticagrelor (109). Cesaro et al. reported the results of a real-world observation study of the long-term use of low dose ticagrelor 1 year after an MI. they reported a rate of 3.9% of MI, and no major bleeding events (110). Piccolo et al. published the rationale and design of the PLINY THE ELDERY trial. It's a pharmacological study looking into the effect of low dose ticagrelor on platelet inhibition compared to the recommended dose in elderly patients with ACS. Platelet reactivity will be determined by VerifyNow (111). The results of this study will give more insight into the possible role of low dose ticagrelor in high risk patients.

Overall, the choice of antiplatelet agent after PCI should be based on a comprehensive approach considering thrombotic risk, bleeding risk, and potential barriers to patient's compliance with treatment. Even though current ACC and ESC guidelines still do not recommend the routine use of platelet function testing or genetic testing in clinical practice, the growing body of evidence suggests that the guidelines will soon reflect the potential value of a guided approach to antiplatelet therapy.

## Author Contributions

AA, YR, DB, KB, ELM, EE, and RJG: substantial contributions to the conception or design of the work, drafting the work or revising it critically for important intellectual content, and provided approval for publication of the content. AA-a and RG agreed to be accountable for all aspects of the work in ensuring that questions related to the accuracy or integrity of any part of the work are appropriately investigated and resolved. All authors contributed to the article and approved the submitted version.

## Funding

Dr. Gumina is supported by NHLBI R01HL127442.

## Conflict of Interest

The authors declare that the research was conducted in the absence of any commercial or financial relationships that could be construed as a potential conflict of interest.

## Publisher's Note

All claims expressed in this article are solely those of the authors and do not necessarily represent those of their affiliated organizations, or those of the publisher, the editors and the reviewers. Any product that may be evaluated in this article, or claim that may be made by its manufacturer, is not guaranteed or endorsed by the publisher.

## Abbreviations

P2Y12, Purinergic receptor type Y: subtype 12
DAPT: Dual antiplatelet therapy
CAD: Coronary artery disease
PCI: Percutaneous coronary intervention
ACS: acute coronary syndrome
ACC: American College of Cardiology
CYP: cytochrome P450
LOF: Loss of function
MACE: major adverse cardiovascular events
RCTs: randomized clinical trials
ADP: adenosine diphosphate
GPIIb/IIIa: Glycoprotein IIb/IIIa integrin
CPIC: Clinical Pharmacogenetics Implementation Consortium
PM: poor metabolizers
IM: intermediate metabolizers
NM: normal metabolizers
RM: rapid metabolizers
UM: ultrarapid metabolizers
TRITON-TIMI 38: Trial to Assess Improvement in Therapeutic Outcomes by Optimizing Platelet Inhibition with Prasugrel–Thrombolysis in Myocardial Infarction—TIMI 38
PLATO trial: PLATelet inhibition and patient Outcome trial
PFT: Platelet function testing
VASP: vasodilator-stimulated phosphoprotein
MAP: Multiplate analyzer
TEG: Thromboelastography
HRP: High platelet reactivity
ADAPT-DES: The Assessment of Dual AntiPlatelet Therapy With Drug Eluting Stents
PRU: Platelet reactivity unit
GRAVITAS, Gauging Responsiveness with A VerifyNow assay—Impact on Thrombosis And Safety ARCTIC: The Assessment by a Double Randomization of a Conventional Antiplatelet Strategy vs. a Monitoring-guided Strategy for Drug-Eluting Stent Implantation and of Treatment Interruption Continuation 1 Year after Stenting
LPR: low platelet reactivity
ANTARCTIC, Assessment of a Normal vs. Tailored Dose of Prasugrel after Stenting in Patients Aged >75 years to Reduce the Composite of Bleeding: Stent Thrombosis and Ischemic Complications
TROPICAL ACS: Guided de-escalation of antiplatelet treatment in patients with acute coronary syndrome undergoing percutaneous coronary intervention
ADPTest: adenosine diphosphate test
IGNITE: Implementing Genomics In Practice
GIANT: Routine CYP2C19 Genotyping to Adjust Thienopyridine Treatment After Primary PCI for STEMI
STEMI: ST-elevation myocardial infarction
PHARM-ACS, Impact of Implementing CYP2C19 Genotype-Guided Antiplatelet Therapy on P2Y12 Inhibitor Selection and Clinical Outcomes in Acute Coronary Syndrome Patients After Percutaneous Coronary Intervention: A Real-World Study in China
PHARMCLO: Pharmacogenomic Approach to Selecting Antiplatelet Therapy in Patients With Acute Coronary Syndromes
POPular Genetics, Cost-effectiveness of Genotype Guided Treatment With Antiplatelet Drugs in STEMI Patients: Optimization of Treatment
TAILOR-PCI: Tailored Antiplatelet Therapy Following PCI
RR: risk ratio
ADAPT: Assessment of Prospective CYP2C19 Genotype Guided Dosing of Anti-Platelet Therapy in Percutaneous Coronary Interventions
IAC-PCI: Individual Applications of Clopidogrel after Percutaneous Coronary Intervention
QALY: quality-adjusted life-years
ICER: incremental cost effectiveness ratio
DM: diabetes mellitus
BMI: body mass index
ABCD-GENE, age, BMI, chronic kidney disease, diabetes mellitus: and genotyping
ESC: European Society of Cardiology
FAST-MI: French registry of Acute ST elevation or non-ST-elevation Myocardial Infarction
DOAC: Direct oral anticoagulant.
